# A Facile Synthesis of Cellulose Nanofibers from Corn Cob and Rice Straw by Acid Hydrolysis Method

**DOI:** 10.3390/polym14204383

**Published:** 2022-10-17

**Authors:** Madhuri Rajanna, Latha Muglihalli Shivashankar, Onkarappa Honnebagi Shivamurthy, Shwetha Uramundina Ramachandrappa, Virupaxappa Shekarappa Betageri, Chandan Shivamallu, Raghavendra Hallur Lakshmana Shetty, Saurabh Kumar, Raghavendra G. Amachawadi, Shiva Prasad Kollur

**Affiliations:** 1 Research Centre, Department of Chemistry, G M Institute of Technology, Davangere 577 006, Karnataka, India; 2 R L Science Institute (Autonomous), Belagavi 590 001, Karnataka, India; 3 Department of Biotechnology and Bioinformatics, School of Life Sciences, JSS Academy of Higher Education & Research, Mysuru 570 015, Karnataka, India; 4 Centre for Biotechnology, Pravara Institute of Medical Sciences (Deemed to be University), Loni 413 736, Maharashtra, India; 5 Kerry Food Center Inc., Beloit, WI 590 001, USA; 6 Department of Clinical Sciences, College of Veterinary Medicine, Kansas State University, Manhattan, KS 66506-5606, USA; 7 School of Physical Sciences, Amrita Vishwa Vidyapeetham, Mysuru Campus, Mysuru 570 026, Karnataka, India

**Keywords:** corn cob, rice straw, nanocellulose, acid hydrolysis, biodegradable

## Abstract

Agricultural residues are suitable to make useful products that can potentially replace the non-biodegradable polymeric materials. In the present work, corn cob and rice S=straw is quantitatively transformed to cellulose by alkali treatment preceded by bleaching. The obtained cellulose is changed into nanocellulose (NC) by the acid hydrolysis method followed by ultrasonication. Further NC was characterized by FTIR to study its functional regions and XRD for crystallinity. Thermal properties have been studied using TGA/DTA. The surface morphology of nanocellulose was done using SEM and TEM. The obtained results revealed remarkable thermal stability, semi-crystalline and fibrous nature of both corn cob and rice straw. The size of the cellulose is in the nanoscale dimension. This work provides the way to utilize corn cob and rice straw as a more useful raw material for many applications.

## 1. Introduction

India is one of the major agricultural and food products producing countries in the world. Most of the agricultural residues can be utilized productively by industries, leading to the production of abundant useful products. All the agricultural residues are lignocellulosic biomass comprised of cellulose, lignin, hemicellulose, pectin, and other components. All these components possess certain physical and chemical properties which can become the base for producing certain products. Usually, agricultural cellulose waste products are referred to as all the parts of a plant, except fruits such as rice husk, corn cob, sugarcane bagasse, rice straw, wheat straw, etc. [1].

Cellulose is the natural and most abundantly available polymer, consisting of a linear chain of β-1, 4 linked D-glucose units [2,3]. Many properties of cellulose depend on its chain length and several glucose units that make up one polymer molecule. The presence of three main polymers—hemicellulose, cellulose, and lignin—can be seen in the cell wall structure of any lignocellulosic biomass. Based on its source, type, and species, the percentage composition of these components varies from one to another [4,5]. About 25% of lignin is present in any dry cellulosic mass in general [6,7,8].

This cellulose fiber-reinforced polymer generally has properties like low density, combustibility, non-toxicity, high tensile strength, low conductivity, and high resistivity [9]. Additionally, the wastes from agricultural biomass and forest residues have great potential to substitute as a feedstock or fuel, which can lead to the production of high value-added materials. The cellulose is converted to nanocellulose (NC) using several physicochemical methods like acid hydrolysis, TEMPO oxidation, and Ionic liquid; however, in this work, the acid hydrolysis method is employed to obtain nanocellulose [10,11,12].

Corn cob and rice straw are agricultural residues; a huge amount of corn cob becomes an agricultural waste soon after the corn pressing [13]. One of the major commercial crops across the globe is maize. Corn cob is the central core of maize. It contains a high concentration of cellulose of 27.71% and hemicellulose of 38.78%. Almost 18% of corn cob is produced from 100 kg of corn ear [14]. During the harvesting time of rice, rice straw is obtained as a residual byproduct. Based on various properties of rice straw, its utilization can be done. Its properties are categorized as physical, thermal, and chemical. Bulk density, thermal conductivity, and heat capacity are the physical properties. Heating value property is relevant with conversion of biomass to energy. Lastly, chemical properties like cellulose, hemicellulose, carbohydrates, and other chemical constituents are the major interests for researchers Corn cob and rice straw are the agricultural residues that are selectively useful for many conventional modes of applications like fodder for animals and firing purposes. However, it is the time to make use of waste material into value-added products [15].

Corn cob and rice straw generate agricultural waste during processing in huge quantities, of which disposal invariably creates environmental burden because most farmers prefer to burn it off. Therefore, its application as raw or modified nanocellulose is highly encouraged as it will reduce the burden of burning-off, resulting in environmental pollution of air and water. It is an inexpensive and environmentally friendly effective adsorbent which has been applied to remediate heavy metals, dyes, and crude-oil polluted water. Both are having wide potential applications in many fields of cosmetics, transdermal drug delivery systems, hydrogels in pharmaceutical preparations, wound healing and wound dressing, aerogels, ophthalmology, packaging, tissue engineering, and biosensor applications, etc., [16].

Based on the previous literature reports, the acid hydrolysis process is employed for the synthesis of nanocellulose to obtain nanocrystals, but this study emphasizes the preparation of nanofibrous and nanofibrils. Many other previous research findings employed 65% of H_2_SO_4_ [17], i.e., higher concentration but presently a lower concentration of 45% is used to prepare nanocellulose [18]. NC obtained is unique with long fibrils and the fibrous nature and size range is even less than 70 nm, with good thermal stability corresponding to previous works.

In the present work, corn cob and rice straw were pre-treated using the alkali and bleaching methods. Further obtained cellulose is treated with acid hydrolysis to obtain nanocellulose. The prepared NC was characterized using FTIR, XRD, SEM, TEM, and TGA/DTA. In this study, a comparative analysis has been done on the NCs and futuristic approaches were detailed.

## 2. Materials and Methodology

### 2.1. Materials

Corn cob and rice straw were collected from local farmers in and around Davangere, Karnataka, India. Acetic acid (CH_3_COOH), sulfuric acid (H_2_SO_4_), sodium chlorite (NaClO_2_), and sodium hydroxide (NaOH) were acquired from SD Fine Chemicals (Location, India). All chemicals were used without any further purification and were of 99% purity. The size and shape of as-prepared material was measured using Transmission Electron Microscopy (TEM-1011, JEOL, Tokyo, Japan). The surface morphology and elemental composition of the obtained material was measured using Scanning Electron Microscope with Energy dispersive X-ray Analysis (Hitachi S3400n, Tokyo, Japan). X-ray diffraction pattern was recorded on a PANalytical X’Pert-PRO (Rigaku Smart Lab). Infrared spectrum of the as-prepared material was recorded using a Perkin Elmer FT-IR spectrometer in the frequency range 4000–500 cm^−1^ (Paragon-1000-FTIR, Buckinghamshire, UK).

### 2.2. Synthesis of Nanocellulose from Corn Cob (CC-NC) and Rice Straw (RS-NC)

The corn cob (CC) and rice straw (RS) fibers was thoroughly cleaned with distilled water. It was compelled into fine powder by drying and sieving. An adequate amount of CC and RW were pre-treated in 5% NaOH solution for about 3 h under constant stirring, at 80 to 100 °C. The acquired celluloses were completely cleaned by washing them repeatedly with the help of distilled water till the effluent was neutral. The enhanced exposure of fibers is promoted by alkali treatment which equips the cellulose for acid treatment and bleaching. The obtained pre-treated husk was exposed to bleaching treatment by using 5% of NaClO_2_ solution for nearly 3 h (80–100 °C) in acidic pH. The residual lignin was excluded by washing repetitively with distilled H_2_O. A neutral pH was achieved by repetitive filtering and washing using deionized water. The obtained cellulose was used for future study by drying it in an oven for about 24 h and was stored in a dry place [19].

The desired quantity of the respective celluloses was combined with 45% *w/v* 50 mL of sulfuric acid separately. The obtained mixtures were hydrolyzed for 60 min at 45 °C under continuous stirring. Then, 100 mL of cold water was added to the reaction mixture to arrest the hydrolysis. The residual slurry was washed by centrifuging it for 10 min repetitively. The resulting supernatant from the sediment was removed and substituted with distilled water. The procedure was followed until the pH of water reached 7 using 2% NaOH. To achieve AH-CCNC and AH-RSNC, the suspensions were ultra-sonicated for about 10 min and subsequently stored at 4 °C in a refrigerator for future use. The total yield of nanocellulose was marked to be 85%.

### 2.3. Characterization

AH-CCNC and AH-RSNC were measured as KBr pellets to gather the Fourier transform infrared spectra (FT-IR) by NICOLET 370 model in the range 4000–400 cm^−1^. Rigaku Miniflex 600 5th gen was used to obtain X-ray diffractogram. The measurement was done with the help of Cu Kβ with a step size of 0.02, and by using Scherer’s formula, the crystallinity index (CI) was estimated. Scanning electron microscopy (SEM) images were recorded on JOEL JSM 6390LV, with an acceleration voltage of 10 kV and a Secondary Electron (SE) detector was used to capture the images. Thermal analysis was done by using Perkin Elmer STA 6000 instrument for Differential Thermal Analysis and Thermogravimetric analysis. Model-Perkin Elmer, diamond TG/DTA, temperature range—ambient +1200 °C, TG measurement range—200 mg, TG sensitivity 0.2 mg, DTA measurement range—1000 mV, DTA sensitivity—0.06 mV, programmable rate 0.01–100 °C/min, sample pan volume-45 mL or 90 mL, single beam temperature range 15 to 900 °C, temperature accuracy −b ± 0.5 °C, thermocouples—PT-PT/Rh (Type R).

## 3. Results and Discussion

### 3.1. Infrared Spectroscopy (FT-IR)

**Figure 1:**
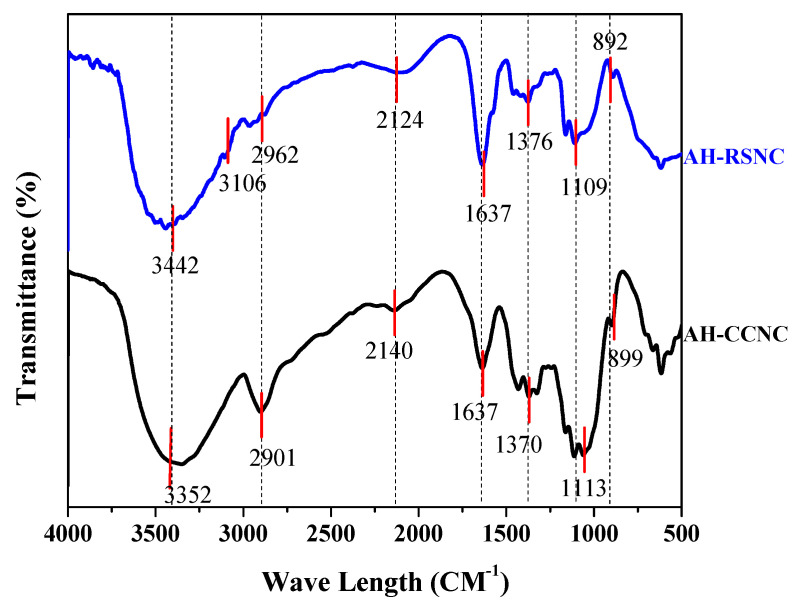
FT-IR of AH-CCNC and AH-RSNC.

To monitor the functional groups, present and understand the physicochemical properties, FT-IR spectroscopy was used. The functional group present in the final product is shown in Figure 1. This image represents the spectra of AH-CCNC and AH-RSNC. From the spectra, it was clearly inferred that both the samples retained the backbone structure of cellulose. The absorption peaks seen in both samples are like each other. The absorption peaks at 3442 cm^−1^ and 3352 cm^−1^ pertain to the CH_2_ groups that are present in the sample. The peaks also show that there exists intermolecular hydrogen bonding attraction between the -CH_2_ groups. The peaks also denote the elongating force experienced by them from -OH groups present. The results also insinuated that there is the presence of the polysaccharide aromatic ring and β-glycosidic linkage. The glucose units registered shown that there was no alteration in the principal structure. Thus, we conclude that the employed procedure does not affect the principal functional groups. The peaks observed at 2140 and 2124 cm^−1^ is attributed to the excessive stretching experienced by the C-C bonds present in AH-CCNC and AH-RSNC. The showcasing of the presence of -CH groups was seen at 2901 and 2962 cm^−1^. The blunt peaks at 1113 and 1109 cm^−1^ denote the abrupt stretching of strong C-O groups present in the sample. The humps at 1370 and 1376 cm^−1^ note the vibrations experienced by the -CH groups present in them. Thus, the employed technique did not affect the principal groups. The peaks at 2901, 2962, and 2923 cm^−1^ correspond to C-H groups.

### 3.2. X-ray Diffraction Analysis

**Figure 2:**
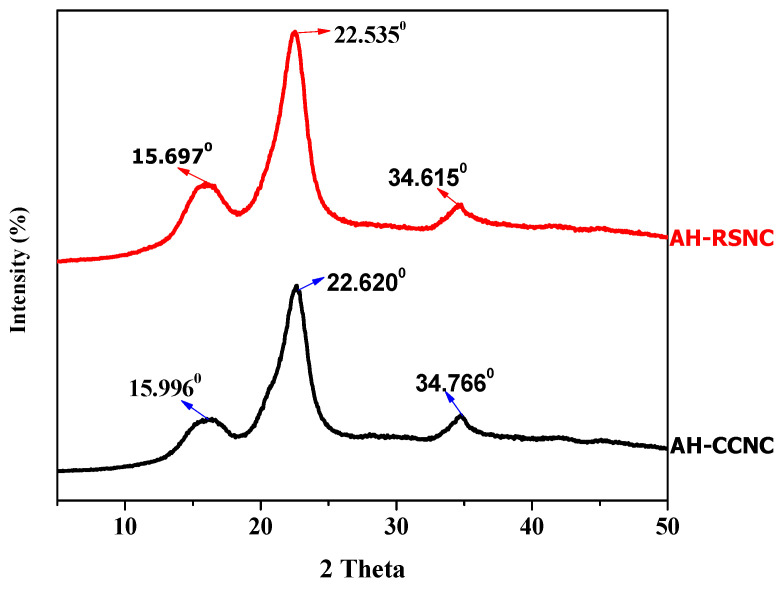
XRD pattern of AH-CCNC and AH-RSNC.

X-ray diffraction method was used to study the crystalline nature of the AH-samples. The X-ray diffractograms of AH-RSNC and AH-CCNC are shown in Figure 2. The intense 2θ values are seen at 22.5350 and 22.6200, representing the intense peaks of AH-RSNC and AH-CCNC owing to its preferred crystal orientation. On a closer investigation, it can be concluded that there is no adverse effect on the principal structure of the cellulose due to ultrasonication. It was also further noticed there was no cavitation effect, thus remarking that acid hydrolysis with ultra-sonication does not affect the innate structure of the cellulose. However, the effect of this is seen in the amorphous regions of the cellulose. Thus, leading to the slight increase in the crystallinity of the sample. The peaks at 15.6970, 15.9960, 34.6150, and 34.6150 show the random arrangement of the crystals. On a closer look, we can conclude the crystallinity index shown by both the samples are very similar [20].

To study the crystalline structure of three NC samples obtained by acid hydrolysis, X-ray diffraction was utilized. It is observed that the samples show the intense peak 2θ at AH-RSNC-22.630 and AH-CCNC-22.690, which corresponds to the structure of Cellulose-I. This concludes that acid hydrolysis with ultra-sonication does not change the principal chemical structure of the cellulose. Besides, there is no selectivity in the cavitation effect when the samples underwent ultra-sonication; however, it affects both amorphous and crystalline regions. Therefore, there is only a slight increase in the crystallinity of AH-RSNC and AH-CCNC, which is chargeable to the distortion of the amorphous regions only because crystalline regions are inert to the chemical treatments [21].

### 3.3. Scanning Electron Microscopy

**Figure 3:**
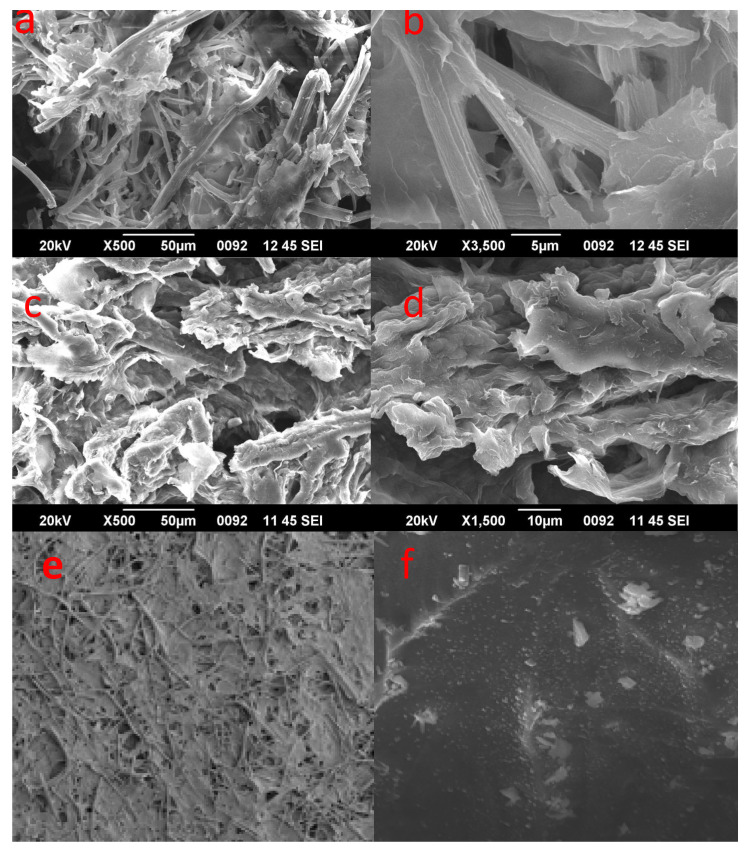
SEM monographs of (**a**,**b**) AH-CCNC, (**c**,**d**) AH-RSNC, (**e**) rice straw cellulose (**f**) corn cob cellulose.

Changes in surface morphologies and topography of the AH samples were studied using SEM images. This led to revealing the clustered long fiber structures of NC. The SEM images shown in Figure 3 indicate strong agglomeration with a fibrous nature and fibrils nature. All the samples were acid-hydrolyzed at 45 °C for 60 min. The morphology of rice straw cellulose (Figure 3e) is observed to have a fiber, showing the potential to be further broken down to individual nano fibers. Corn cob cellulose of untreated raw material is observed to have a uniform, smooth surface without any distortion compared to the treated sample [22]. It is evident from the monographs in Figure 3a,b that the size of the cellulose has been clearly reduced to nano terms, free from lignin and has a low aspect ratio.

Figure 3a,b shows the images of AH-CCNC. It is evident from the monographs that the obtained nanocellulose is free of lignin and its size has been reduced to the nano-level. Furthermore, it can be noted that the sample has a low aspect ratio. There are lesser impurities observed due to the removal of hemicellulose and lignin along with the cluster of micro-fibrils in huge numbers. It has a non-uniform surface with an irregular cross-section. There is the presence of long fibrils which are tangled with the smaller ones, creating a webbed assembly. However, it can be marked that the size of the fibers is at the nano-level.

Figure 3c,d shows monographs of the AH-RSNC sample. The particles have a more non- uniform and rough surface, with no presence of impurities, hence lacking any valley formation. The reason is the repetitive washing of the nanocellulose and ultra-sonication. The size distribution increased commendably because of the subtraction of the amorphous regions in the crystallinity chain. It should be noted that, even though CC and RS were hydrolyzed in similar conditions and temperatures, the resulting yield is slightly different in the surface study. It is stated that all the samples were successfully converted in nano terms with the aid of the employed method which gave commendable results. The two samples have a different shape—fibrils and fibrous nature—owing to the nanoscale [23].

### 3.4. Transmission Electron Microscopy

**Figure 4:**
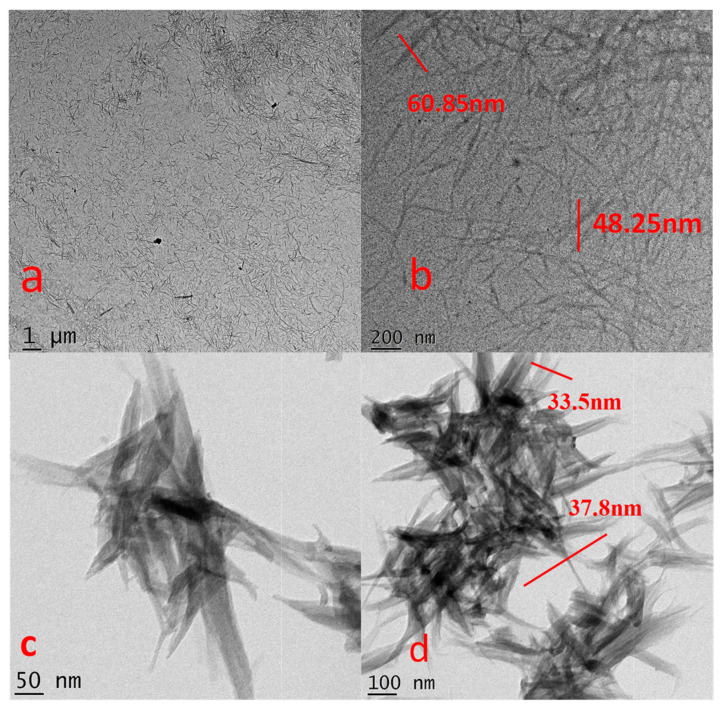
TEM images of (**a**,**b**) AH-CCNC and (**c**,**d**) AH-RSNC.

To study the surface morphologies and the internal arrangement of the prepared nanocelluloses, TEM analysis was used. Figure 4a,b and Figure 4c,d are the TEM images of AH-CCNC and AH-RSNC, respectively. It is observed that, the formation of elongated chains falls in nanoscale. The nano fibers obtained are finely separated from one another.

They have several nano-fibrils that are rod-like structures. The size of the prepared nanocelluloses of AH-CCNC and AH-RSNC (highlighted with red lines) is in the range of 48–69 nm, 33–38 nm, respectively, thus, proving that they are in nanoscale. TEM images clearly reveal the presence of strong agglomeration present in the sample. Since the sample has overcome the strong hydrogen bond influence, we can notice the sharp ends. This is also because of the repulsions experienced as the surfaces are negatively charged.

### 3.5. Thermogravimetric Analysis and Differential Thermal Analysis (TGA/DTA)

**Figure 5:**
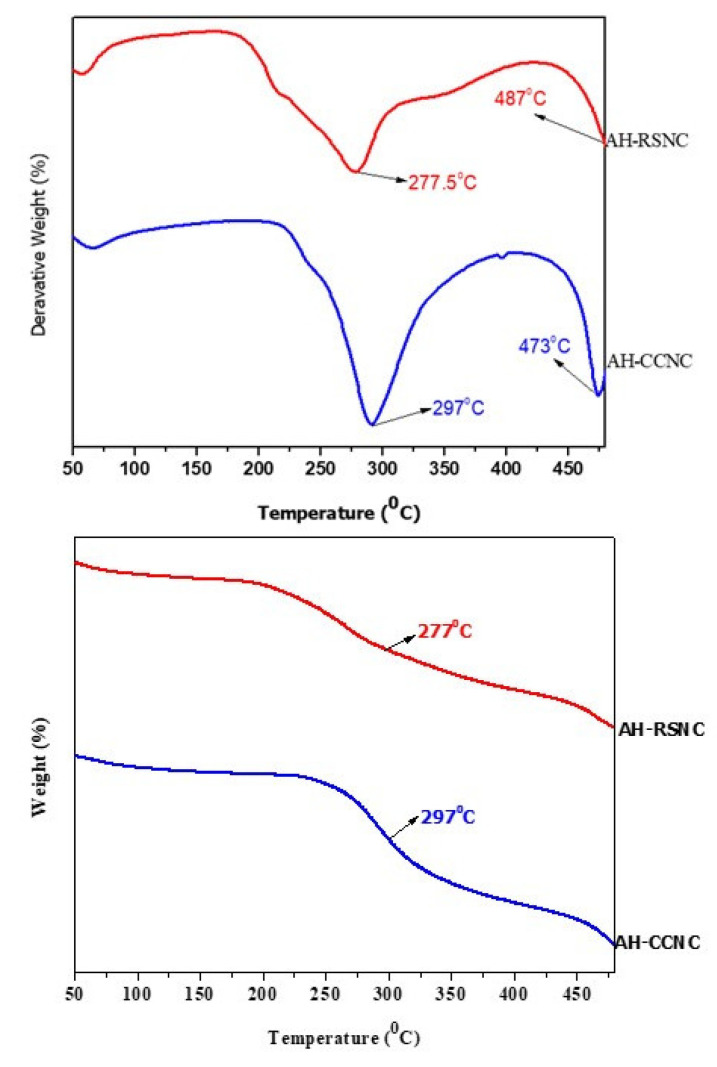
(**top**) DTG thermogram of AH-CCNC (blue) and AH-RSNC (red). (**bottom**) DTA thermogram showing degradation curves for AH-CCNC (blue) and AH-RSNC (red).

The morphological and thermal stability of the synthesized samples AH-CCNC and AH-RSNC was studied with the help of Derivative Thermogravimetric (DTA) and Thermogravimetric Analysis (TGA). The TGA and DTA curves obtained are shown in Figure 5. The evident weight loss is seen in the sample because of the evaporation of all the water present in the sample. The obtained degradation temperature was found to be 277.5 °C and 297 °C for AH-RSNC and AH-CCNC, respectively. This can be seen in the initial fall in the graph. AH-CCNC shows a sudden drop in its weight at around 297 °C for AH-CCNC and at 277 °C for AH-RSNC. This is seen because of the breakage of all the glycosidic bondages with the de-polymerization of the cellulose.

Out of the two synthesized materials, AH-CCNC shows a better thermal stability when compared to AH-RSNC. The deep curves seen in the graphs are due to the hydrolysis employed using sulfuric acid [24,25].

## 4. Conclusions

To sum up, the present work is entitled to give detailed information on nanocellulose (NC) extracted from corn cob and rice straw, which served as the starting material. The acid hydrolysis (45% W/V) method was employed to cellulose to achieve nanocellulose. The results obtained in the present work focus on the physical property of the end product, including its morphological change. The properties based on its morphology were studied with the aid of analytical methods. The characteristic peaks shown by AH-CCNC and AH-RSNC clearly state the crystallinity of the cellulose after undergoing acid hydrolysis treatment. Further, FT-IR spectra show that the presence of principal functional groups is like that of cellulose peaks. The crystallinity exhibited by both the samples are similar. It is also noticed where the fibrous and fibrillar regions are clearly seen from the SEM monographs. TEM monographs show the nanoscale range of both CCNC and RSNC are less than 70 nm when compared to previous studies. AH-CCNC seems to excel in the aspects of thermal stability and aspect ratio, with increased stability of dispersion because of its excellent absorption efficiency. Thus, it can be stated that AH-CCNC serves as a best fit for future applications as it comes with excellent physical and chemical properties tied with remarkable dispersive property. This gives a new way to make use of the naturally available biomass in nanomaterial applications. The obtained nanocellulose can be effectively used in the production of thin films with antimicrobial activity, which can be used in water purification, pharmaceuticals, nanochip production, biodegradable nanocomposites, and many more. Between the two synthesized NCs, AH-CCNC serves as the best material for futuristic use when compared to others.

## Data Availability

Not applicable.
